# Benthic fluxes of dissolved organic carbon from gas hydrate sediments in the northern South China Sea

**DOI:** 10.1038/srep29597

**Published:** 2016-07-19

**Authors:** Chia-Wei Hung, Kuo-Hao Huang, Yung-Yen Shih, Yu-Shih Lin, Hsin-Hung Chen, Chau-Chang Wang, Chuang-Yi Ho, Chin-Chang Hung, David J. Burdige

**Affiliations:** 1 Department of Oceanography, and Asia-Pacific Ocean Research Center, National Sun Yat-sen University, Kaohsiung, 80424 Taiwan; 2 Department of Applied Science, R.O.C Naval Academy, Kaohsiung 81345, Taiwan; 3 Institute of Undersea Technology, National Sun Yat-sen University, Kaohsiung, 80424 Taiwan; 4 Department of Ocean, Ocean, Earth and Atmospheric Sciences, Old Dominion University, Norfolk, VA, 23529 USA

## Abstract

Hydrocarbon vents have recently been reported to contribute considerable amounts of dissolved organic carbon (DOC) to the oceans. Many such hydrocarbon vents widely exist in the northern South China Sea (NSCS). To investigate if these hydrocarbon vent sites release DOC, we used a real-time video multiple-corer to collect bottom seawater and surface sediments at vent sites. We analyzed concentrations of DOC in these samples and estimated DOC fluxes. Elevated DOC concentrations in the porewaters were found at some sites suggesting that DOC may come from these hydrocarbon vents. Benthic fluxes of DOC from these sediments were 28 to 1264 μmol m^−2 ^d^−1^ (on average ~321 μmol m^−2 ^d^−1^) which are several times higher than most DOC fluxes in coastal and continental margin sediments. The results demonstrate that the real-time video multiple-corer can precisely collect samples at vent sites. The estimated benthic DOC flux from the methane venting sites (8.6 × 10^6 ^mol y^−1^), is 24% of the DOC discharge from the Pearl River to the South China Sea, indicating that these sediments make an important contribution to the DOC in deep waters.

Dissolved organic carbon (DOC) in seawater is a major component of the marine carbon cycle and is the second largest pool of carbon in the ocean after the pool of dissolved inorganic carbon. DOC had originally been thought to be generated primarily by the activity of marine organisms in surface waters and then transported to deep waters^1^,^2^. Some researchers have also reported that benthic fluxes of DOC from continental margin sediments may be responsible for a significant fraction of the DOC input to the deep ocean^3^,^4^,^5^. The magnitude of this flux may also be of the same order as the riverine input of DOC, although DOC in the deep ocean shows little evidence of a terrestrial signature, suggesting that much of this riverine DOC is rapidly remineralized (for a recent review see Dittmar and Subbins^6^). Recently, petroleum seeps, cold seeps and mud volcanoes have also been reported to contribute DOC to the oceans^7^,^8^.

Recent research reveals that mud volcanoes, gas hydrates and cold seeps are widely distributed in the northern South China Sea (NSCS, Fig. 1) offshore of southwestern Taiwan^9^,^10^,^11^. The region is in the frontal area of the accretionary wedge of the Luzon Arc and the China passive continental margin subduction–collision system^9^. The active discharge rates coupled with the episodic escape of warm fluids may allow the gas phase to persist and to reach the water column, where it dissolves and generates a large methane plume^12^. A series of fold-and-thrust structures are the dominant features in this accretionary wedge province^9^. These tectonic-driven structures may represent good conduits for gases and liquids venting upwards into the ocean. Numerous active submarine mud volcanoes, gas seepage and pockmarks have been observed in waters off southwest Taiwan^10^,^13^, and researchers have documented that both bottom waters and sediment pore waters at these mud volcanoes exhibit high methane concentrations, revealing gas venting features in these structures^10^,^14^,^15^. A thermal fluid may migrate from the anticline structure to the ridge crest, and then rise up to the seefloor along faults or fissures^13^. Some researchers have also measured high dissolved organic carbon in the pore waters in similar geological settings^7^,^8^,^16^ suggesting that such a DOC flux may also be important from mud volcanoes and gas seeps in the South China Sea. While these mud volcanoes and gas seeps may play a significant role in providing dissolved organic carbon (DOC) to the ocean, to date only a few studies have estimated deep water DOC in the NSCS. To better understand the effects of mud volcanoes and gassy sediments on the concentration of DOC in the deep ocean, we collected bottom water and porewater samples to measure DOC concentrations using a real-time video multiple corer (V Corer) in the NSCS in 2014, and used these results to evaluate the DOC flux across the sediment-water interface at these sites.

The use of a real-time video multiple-corer to take samples from gas hydrate sediments offers distinct advantages over other types of sampling devices (e.g., piston or gravity cores), since it provides direct visual evidence that one is sampling a venting site. Scientists have been studying gassy sediments in the South China Sea for over 10 years (see, for example, Yang *et al*.^17^), but they have seldom observed high methane or DOC fluxes due to the use of traditional piston or gravity coring without real-time video.

## Results and Discussion

### DOC in bottom waters and porewaters

**Figure 1:**
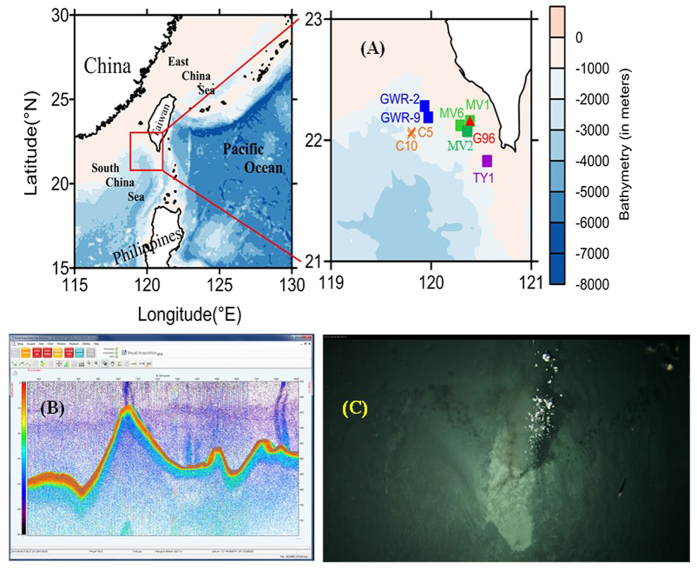
(**A**) Sampling locations in the northern South China Sea (The map was created using Surfer software v.12 Surfer (Golden Software) http://www.goldensoftware.com/home/terms-of-use). (**B**) Screenshot of the BioSonics echosounder onboard the R/V OR5 detecting bubbles plumes (methane bubble: blue chimney, seafloor: orange curve in the bottom) in the water column. The hydroacoustic data was collected using BioSonics Visual Acquisition software version 6.0. (**C**) Screenshot of the video was taken in the study area by the deep sea towed vehicle ATIS (Abyss Twisted-pair Imaging System) operated by the National Sun Yat-sen University.

**Figure 2:**
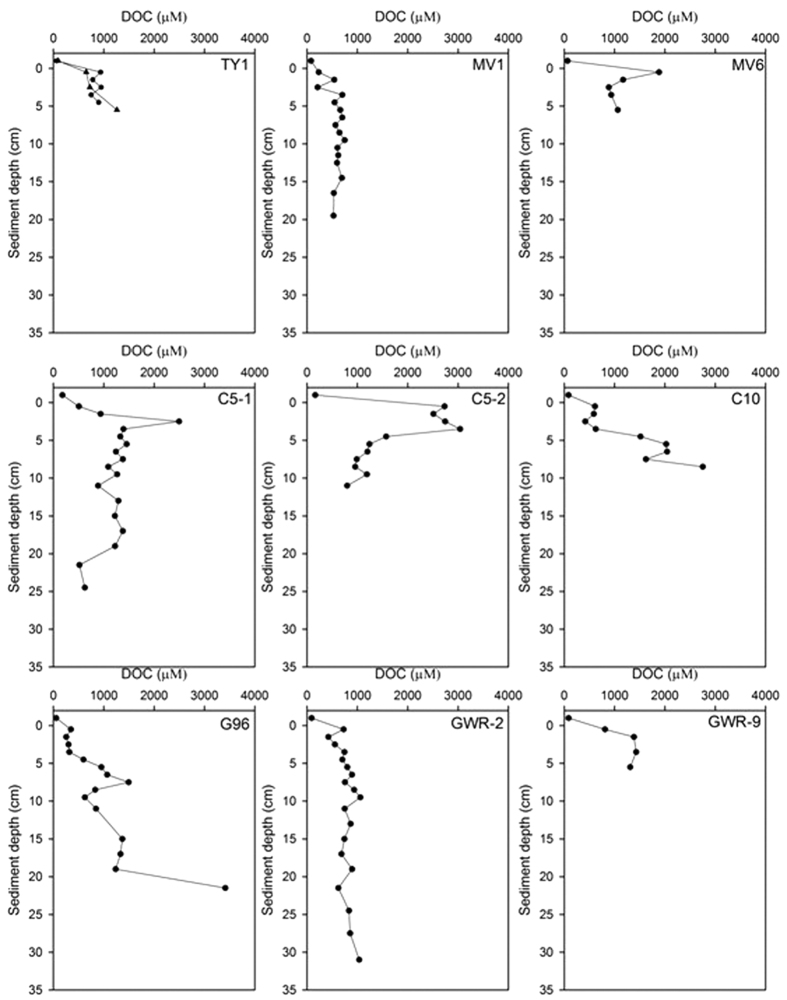
Depth profiles of DOC in bottom waters (above the zero position of y-axis) and porewaters at different stations from the northern South China Sea. At station TY1, circles and triangles represent sampling times in June and August 2014, respectively.

DOC concentrations in bottom water and sediment porewaters are reported in Table 1 and porewater profiles are shown in Fig. 2. Concentrations (53 ~ 178 μM) of DOC in the bottom waters were several times lower than those in porewaters (232 ~ 2733 μM). At most sites, DOC in the porewaters increased continuously with increasing depth, although one point (MV6 and C5-1) and multi-point sub-surface maxima (C5-2) were seen in three cores (Fig. 2; note porewater DOC profiles at two background sites are not shown here). The lowest bottom water DOC concentrations were observed at background site BKG-1 and MV6 (Table 1). The DOC concentration in the bottom water at another background site is 107 μM, higher than other regions, suggesting that it could be a combination of high DOC emission water and deep sea water in the NSCS. The maximum and minimum DOC concentrations in porewaters occurred at sites C5-2 and MV1. At TY1, there were two sampling cruises in June and August, respectively. The sampling positions were similar, and there was very good agreement between the profiles, suggesting that site TY1 has stable DOC concentrations and fluxes. It is noted that “duplicate” cores from sites C5 and GWR show differences because of differences in the specific sampling locations and water depths (for details see Table 1 and Fig. 1).

The vertical gradients of porewater DOC profiles at mud volcano stations TY1, MV1, MV6 and GWR-2 (not a mud volcano) are similar, with maximum porewater DOC concentrations of ~1000 μM. In contrast, at other stations where there are cold seep vents and hard sediments with gas bubbles pore water DOC concentrations are higher at depth in the sediments (up to 2000 to 3000 μM). These differences may reflect the fact that porosity and sediments characteristics at distinct geographic settings may affect porewater DOC profiles.

### Benthic DOC flux

The estimated benthic fluxes of DOC from these sediments in the NSCS ranged from 28 to 1264 μmol m^−2 ^d^−1^ (average, ~321 μmol m^−2 ^d^−1^) (Table 2) which is higher than values observed here at the background sites (8 ~ 10 μmol m^−2 ^d^−1^) and recently reported values (9 ~ 196 μmol m^−2 ^d^−1^) from sites “near” methane venting sites in the NSCS^18^. As described earlier, we used a real-time video corer to precisely collect sediments and bottom waters near methane venting sites, while Ho and Hung^18^ used a multiple-corer to take samples near methane venting sites based solely on sonar images.

To begin to examine the possible sources of the DOC in these benthic fluxes, we note that the POC flux to sediments in this region is ~4 μmol m^−2 ^d^−1^ 19. If all of this POC is remineralized in the sediments, only a fraction (generally less than ~10–20%) will likely be transformed during remineralization processes into DOC that escapes the sediments as a benthic flux^20^. This observation demonstrates that DOC produced by other processes associated with mud volcanoes or gassy sediments are important sources of these DOC benthic fluxes to the deep water in the NSCS. Episodic weather events such as typhoons may result in transient pulses of fresh organic matter to these sediments^21^,^22^,^23^,^24^ that may enhance the downward flux of POC to these sediments above that predicted by these more traditional particle flux measurements. However, the magnitude of such events (and their impact on benthic DOC fluxes) is difficult to quantify.

Our results suggest that the DOC benthic flux to deep water around vent sites in the NSCS can be large, but what is the overall significance of these fluxes? The mean benthic flux in the study area (~321 μmol m^−2 ^d^−1^; Table 2) is ~0.9 to 6 times higher than reported values (50 ~ 350 μmol m^−2 ^d^−1^) in most continental margins sediments, and ~8 ~ 9 times lower than fluxes from estuarine sediments (up to 3000 μmol m^−2 ^d^−1^, Table 3). While benthic DOC fluxes from NSCS vent sites are also significantly lower than fluxes from methane hydrate-bearing seeps in the eastern North Pacific Ocean^8^ (93,000 ± 66,000 μmol m^−2 ^d^−1^, Table 3) the uncertainty of these latter fluxes is quite large.

Note however, that at sites where there is active bubbling of methane out of the sediments, the diffusive fluxes calculated here with eqn. (1) may under-estimate the actual total benthic flux several fold, since gas bubbles maintain open bubble tubes that enhance diffusive transport above that predicted by a simple one-dimensional diffusive flux calculation^25^,^26^. In Cape Lookout Bight, NC (USA), a coastal embayment, seasonal formation of gas bubbles in these sediments enhances the diffusive fluxes of all solutes by ~3-fold when active bubble ebullition occurs^27^. Since this flux enhancement is presumably a function of the rate of sediment gas bubbling, in other sites, such as those we have studied, this enhancement of the diffusive benthic flux may be even greater, and will clearly require further study.

According to the survey of chirp sonar sub-bottom profile and seismic reflection profiles in the NSCS^9^,^28^, mud volcanoes can be grouped into four main clusters in the accretionary wedge province. Each cluster contains a few to more than 10 submarine mud volcanoes. Chen *et al*.^13^ estimated the size of mud volcanoes MV1 and MV2 using high-resolution sub-bottom profiles and the multibeam bathymetry. They reported that MV1 has a diameter of about 1500 m and a height of 100 m while MV2 is about 880 m long and 75 m high^13^. The estimated areas of MV1 and MV2 are approximately 1.76 and 0.50 km^2^ (on average 1.18 km^2^), respectively. Chiu *et al*.^28^ reported that at least 76 gassy sediments and mud volcanoes groups have been identified in the selected study area (water depth 200 ~ 1500 m) in the southwestern Taiwan.

Using this average area (1.18 km^2^) we next assume that the benthic DOC flux around each mud volcano varies from the highest value (516 μmol m^−2 ^d^−1^ at MV6) at the center of the mud volcano to the background level (~9 μmol m^−2 ^d^−1^) at the edge of the mud volcano. The average benthic DOC flux from one mud volcano will then be 263 (=(516 + 9)/2) μmol m^−2 ^d^−1^ and the integrated flux from a single mud volcano will be 310 mole d^−1^ (=1.18 km^2^ × 263 μmol m^−2 ^d^−1^). The total benthic DOC flux to the ocean DOC pool in the NSCS study area (76 locations) is then 8.6 × 10^6 ^mol y^−1^ from mud volcanoes and gassy sediments. We believe that the DOC flux at MV6 (348 μmol m^−2 ^d^−1^) is a conservative value that is close to the mean value (321 μmol m^−2 ^d^−1^) in the study area. However, this estimate has some uncertainties since we are not sure if the DOC flux from center to the edge of the mud volcano decreases linearly and if DOC fluxes from other gassy sediments are similar to what is seen at mud volcanos. Such benthic DOC fluxes definitely require further study.

This integrated benthic DOC flux is approximately 24% of the annual DOC flux (36 × 10^6 ^mol y^−1^) from the Pearl River, the largest river that drains into the South China Sea^29^. If we consider the effect of active methane bubbling (by up to ~3-fold) on the diffusive fluxes of DOC in the study area, the annual benthic DOC flux may represent almost 70% of the DOC flux from the Pearl River to the South China Sea. This clearly suggests that the benthic flux of DOC from gas hydrate sediments is an important source of DOC to the South China Sea. Hsu *et al*.^30^ reported that gas plume emissions may be amplified by tidal exchange in the SCS suggesting that the DOC flux may also be influenced by tidal exchange, especially during periods of high tide. We believe that there are more gassy sediments or mud volcanoes in deep waters (>1500 m) that have not been explored in the NSCS.

### Sources of DOC in bottom waters and potential mechanisms affect the DOC benthic flux

Since POC remineralization in sediments appears to be a minor source of the DOC found in these sediments (and escaping the sediments as a benthic flux), we suggest that this DOC may be the direct result of other processes associated with the venting sites. One possible source is thermogenic DOC production in association with production of methane and other hydrocarbons^10^. However, it appears that much of the emitted methane here has a biogenic source based on it δ^13^C value (−69 ± 5%; n = 4) and the high molecular ratio of methane, ethane and propane 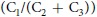
10.

Another possibility is that the emitted methane is partially transferred (or oxidized) to dissolved inorganic carbon or DOC in association with bacterial anaerobic oxidation of methane^8^. Recently Wang *et al*.^31^ observed lighter δ^13^C values (−0.98% to −6.21%) for benthic foraminifera (*Uvigerina proboscidea)* at a methane vent site (e.g. station C5) and heavier δ^13^C values (−0.40% to −0.86%) at the background sites suggesting that benthic foraminifera take up isotopically light carbonate and bicarbonate from the deep sediments. Furthermore, Hung^32^ reported depth profiles of methane concentrations in gas hydrate sediments that increase with increasing depth, suggesting that methane in the sediment is consistently emitted to the deep water, and oxidized in the surface sediments. We did not measure the carbon isotopic composition of other sediment and pore water constituents, but the δ^13^C values in benthic foraminifera and the methane depth profiles in the sediments strongly support our suggestion that some of methane emitted from these gas hydrate sediments is oxidized to DIC or DOC^8^. Using such an approach Pohlman *et al*.^8^ observed that fossil methane accounts for 28% of the DOC in waters overlying gas hydrate bearing seeps in the northeastern Pacific Ocean. Coffin *et al*.^33^, also using carbon isotopes, suggested that emitted methane contributed 7 ~ 71% of DOC in the southern Pacific Ocean near New Zealand. Furthermore, the carbon derived from this methane is much older (depleted in ^14^C), and more depleted in ^13^C than background DOC^8^. According to Liu *et al*.^9^, the study area in the NSCS could be highly associated with known gas fields on the continental shelf and on land in southern Taiwan. The former is likely a normal fault-block trap with thermogenic gas in Oligocene to Miocene sandstones^34^ and the latter is a shallow structural and stratigraphic trap containing biogenic gas in Pleistocene sandstones^35^. Based on these studies, we believe that DOC migration or emissions that accompany methane in this region may partially come from aged carbon in deep sediments, suggesting that such processes in the South China Sea may significantly contribute to aged DOC in the deep ocean.

### Implications of benthic DOC fluxes into deep water in the NSCS

As described in the introduction, the northern South China Sea is in the frontal zone between the Luzon Arc and the China passive continental margin subduction–collision system^9^,^27^, and many active mud volcanoes and gassy sediments have been identified in waters (<1500 m) near southwestern Taiwan^9^,^10^,^13^. Our results^10^ reveal that dissolved organic carbon, from a biogenic source in these sediments, is added to the deep waters here in conjunction with geo fluid migration or gas emission^8^. However, as noted above we have little information about the composition and reactivity of this DOC. Furthermore, the net benthic DOC flux we have estimated here is likely an underestimate because the values estimated here are based on a limited number of sampling locations at gassy sediments or mud volcanoes. We believe that intensive temporal and spatial sampling will better increase our understanding of benthic DOC flux from these types of sediments.

## Conclusions

Benthic DOC fluxes from mud volcano and gassy sediments in the NSCS were approximately 28 and 1264 μmol m^−2 ^d^−1^, respectively, which is one order of magnitude higher than the background level from other sediments in the NSCS and higher than the global average flux from continental margin sediments. The elevated benthic DOC fluxes we determined reveal that the real-time multiple-corer can be used to more precisely obtain sediments and bottom seawater samples near methane seeping vents. The benthic DOC flux from mud volcanoes, either from thermogenic or biogenic sources, may be an important source of DOC to the deep-sea DOC pool in the northern South China Sea as well as the deep ocean in general.

## Methods and Materials

Six expeditions were conducted in the northern South China Sea (NSCS) offshore southwestern Taiwan by R/V Ocean Researcher 5 in 2013 and R/V Ocean Researcher III in 2013 and 2014 (Fig. 1). Chen *et al*.^13^ used a remotely operated vehicle (ROV) and reported that there are numerous, up to ~10’s, venting holes at one mud volcano site in the NSCS. Roughly, the characteristics of the sampling locations can be indentified based on their methane bubble images and sediment nature, and include mud volcanoes (MV), hard sediments with gas bubble images (GWR), and cold seep vents (C). There is one special location (TY), also a mud volcano too, named in memory our colleague Prof. Tsanyao Yang.

In the cruise conducted by R/V OR5 in 2013, we only searched for the detailed positions of gas fluid bubble plumes and took seafloor photos (Fig. 1), but did not take samples using Video-corer (V-corer). On the cruises with R/V OR3 in 2013 and 2014, we searched for venting sites using a fishery echo sounder (model EK60) until we observed acoustic images of bubbles plumes on the computer monitor (Fig. 1). After several scans by the echo sounder, we obtained an accurate venting position and then deployed our V-corer. The V-corer was composed of a real-time video camcorder and a multiple corer, built by Underwater Mechatronics Lab at National Sun Yat-sen University. The V-corer was slowly (~1 m/s) deployed on the top of a venting position at either mud volcano or cold seep sites. After seeing a clear bubble image in the bottom with the V-corer, the V-corer was triggered to take bottom water and surface sediments simultaneously (see the attached video in supplemental material). We also took samples from locations without any bubble image region, defined as background sites (BKG-1 in 2013 and BKG-2 in 2014).

Bottom water was collected to clear ampoules and stored frozen until analysis. Sediment cores were sectioned at 1-cm intervals and the sediment slices were centrifuged at 4000 rpm for 15 min. Porewaters were collected into precombusted 10-ml glass vials after filtration through glass fiber syringe filters (GF/F; Whatman) and stored frozen until analysis. Concentrations of DOC in bottom water and porewaters were determined by a high temperature catalytic oxidation method using a Shimadzu TOC-5000. The benthic DOC flux between bottom water and surface sediment was calculated using Fick’s First Law after Berner^36^. Briefly, the benthic DOC flux (J) was calculated by the following equation.
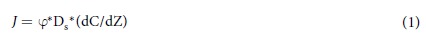
where φ is the sediment porosity, D_s_ is a bulk sediment diffusion coefficient corrected for tortuoisity (*θ*^2^) and dC/dZ is the DOC concentration across the sediment-water interface. Additional details about these calculations are presented in Table 2.

## Additional Information

**How to cite this article**: Hung, C.-W. *et al*. Benthic fluxes of dissolved organic carbon from gas hydrate sediments in the northern South China Sea. *Sci. Rep.* **6**, 29597; doi: 10.1038/srep29597 (2016).

## Supplementary Material

Supplementary Information

Supplementary Video

## Figures and Tables

**Table 1:** Sampling information and concentrations of DOC between bottom water and surface sediments in the northern South China Sea.

**Cruise No**	**Sampling time**(**mm/dd/yy**)	**Station**	**Lat**. (**N**)	**Lon**. (**E**)	**WD** (**m**)	**Core** (**cm**)	**DOC**^**a**^ (**μM**)	**DOC**^**b**^ (**μM**)
1693	06/20/2013	BKG-1	22°15′	120°24′	250	73	52.9	230^c^
1815	11/27/2014	BKG-2	22°15′	120°13′	750	29	106.6	134
1775	06/26/2014	TY1	21°49′	120°33′	400	8	94.7	650
1791	08/27/2014	MV1	22°09′	120°23′	367	21	77.2	232
1791	08/28/2014	TY1	21°49′	120°33′	368	5	62	937
1806	10/29/2014	C5-1	22°04′	119°48′	1448	26	177.5	505
1806	10/29/2014	C5-2	22°02′	119°48′	1354	12	161.3	2733
1806	10/30/2014	C10	22°03′	119°48′	1313	9	85.5	610
1815	11/27/2014	MV6	22°07′	120°17′	668	6	54.7	348
1815	11/27/2014	G96	22°09′	120°23′	385	23	66.6	1883
1823	12/23/2014	GWR-2	22°16′	119°56′	1089	31	88.3	727
1823	12/23/2014	GWR-9	22°11′	119°58′	900	9	87.6	801

Note: WD-water depth, DOC^a^: concentration of DOC in bottom water, DOC^b^: porewater (0 ~ 1 cm), c: sample at 5 cm.

**Table 2:** Detailed parameters for estimating DOC fluxes in gas hydrate-rich sites in the northern South China Sea*.

**Cruise**	**Station**	**Depth** (**cm**)	**DOC** (**μM**)	**Porosity** (**φ**)	**D**_**0**_ (**10**^**−6**^ **cm**^**2**^ **s**^**−1**^)	**θ**^**2**^	**D**_**S**_ (**10**^**−6**^ **m**^**2**^ **d**^**−1**^)	**dC**/**dZ** (**μM**/**m**)	**DOC flux** (**μmol m**^**−2 **^**d**^**−1**^)
1693	BKG-1	bw	52.9	0.50	1.585	2.39	5.74	3533	10.1
		5	229.5						
1815	BKG-2	bw	106.6	0.50	1.585	2.40	5.71	2710	7.7
		1	133.7						
1775	TY1	bw	94.6	0.50	1.585	2.41	5.69	55576	156.6
		1	650.4						
1791	MV1	bw	77.2	0.39	1.585	2.90	4.72	15442	28.1
		1	231.6						
1791	TY1	bw	62.0	0.50	1.585	2.41	5.69	87522	246.5
		1	937.2						
1806	C5-1	bw	177.5	0.63	1.585	1.93	7.08	32733	145.3
		1	504.8						
1806	C5-2	bw	161.3	0.66	1.585	1.84	7.46	257149	1263.9
		1	2732.8						
1806	C10	bw	85.5	0.63	1.585	1.94	7.06	52412	231.3
		1	609.7						
1815	G96	bw	54.6	0.58	1.585	2.10	6.51	29307	110.0
		1	347.7						
1815	MV6	bw	66.6	0.50	1.585	2.40	5.71	181680	516.1
		1	1883.4						
1823	GWR-2	bw	88.3	0.62	1.585	1.97	6.95	63870	273.3
		1	727.0						
1823	GWR-9	bw	87.6	0.54	1.585	2.23	6.13	72259	239.3
		1	810.2						

bw: bottom water.

D_0_ is the free solution diffusion coefficient (=50 cm^2^/yr; Komada *et al*.^5^), while D_s_ is the bulk sediment diffusion coefficient corrected for sediment tortuosity (*θ*^2^) according to
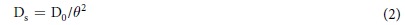

where θ^2^ is estimated using the modified Weissberg relationship (Boudreau^37^),
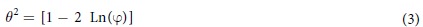

For all sites except BKG-1 the DOC concentration gradient (dC/dz) was calculated as the difference between the bottom water and porewater concentration at 1 cm divided by 1 cm. At BKG-1 a pore water sample at except at 5 cm was used and this concentration difference was divided by 5 cm. With all of these quantities, the benthic DOC flux was calculated with eqn. (1).

**Table 3:** A summary of benthic DOC fluxes from different regions around the world.

**Researchers**	**Study area**	**Water depth** (**m**)	**DOC flux** (**μmol m**^**−2 **^**d**^**−1**^)
Hung *et al*. (this study)	South China Sea	367 ~ 1448	28 ~ 1264
Heggie *et al*.^38^	E. Pacific	3100	102
Bauer *et al*.^39^	NE Pacific	4100	130
Hulth *et al*.^40^	Wedell Sea	2514	577
Hulth *et al*.^40^	Wedell Sea	316 ~ 494	102 ~ 544
Otto & Balzer^41^	NE Atlantic	4805	100
Alperin *et al*.^42^	N Atantic	300 ~ 1000	80
Burdige *et al*.^3^	NE. Pacific	2144 ~ 3595	100 ~ 720
Burdige^43^	estuarine to deep water	10 ~ 3500	100 ~ 3000
Papadimitriou *et al*.^44^	E. North Atlantic	1100 ~ 3500	250 ~ 440
Lahajnar *et al*.^45^	Arabian Sea	3190 ~ 4420	60 ~ 224
Lahajnar *et al*.^45^	NE Atlantic	4500 ~ 4800	47 ~ 121
Pohlman *et al*.^8^	NE Pacific	856 ~ 1309	93000 ± 66000
