# Supplementary material for: Benthic fluxes of dissolved organic carbon from gas hydrate sediments in the northern South China Sea
Source: Sci Rep. 2016 Jul 19;6:29597. doi: 10.1038/srep29597 (PMC4949426; doi:10.1038/srep29597)
Supplement: Supplementary Information [file srep29597-s1.pdf]

Benthic fluxes of dissolved organic carbon from gas hydrate sediments in the northern South China Sea

Chia-Wei Hung[1], Kuo-Hao Huang[1], Yung-Yen Shih[1,2],Yu-Shih Lin[1], Hsin-Hung Chen[3], Chau-Chang Wang[3], Chuang-Yi Ho[1], Chin-Chang Hung[1,4*], and David Burdige[4]

1. Department of Oceanography, and Asia-Pacific Ocean Research Center, National Sun Yat-sen University, Kaohsiung, 80424 Taiwan.
2. Department of Applied Science, R.O.C Naval Academy, Kaohsiung 81345, Taiwan.
3. Institute of Undersea Technology, National Sun Yat-sen University, Kaohsiung, 80424 Taiwan.
4. Department of Ocean, Ocean, Earth and Atmospheric Sciences, Old Dominion University, Norfolk, VA, 23529 USA

Video legends

When the V-corer observes bubbles coming from a sampling site, it is deployed and trigged to take both a bottom water sample and surface sediment cores. The video shows that the real-time V-corer can collect such samples from gassy sediments at vent sites. The timeline below describes the sequence of observations in this video.

Time	Description
0:00	One can see the Video-corer (V-corer) with a reflection mirror (on the left of the multiple corer) taking a real-time image of the V-corer frame (black color) and core tubes.
0:13	As the V-corer gets closer to the sediments, one can begin to see methane bubbles coming out of the gassy sediments and rising upwards.
0:18	As the sediment surface becomes more clearly visible, one can see the pockmarks on the surface due to bubble ebullition from the sediments.
0:21	Landing of the V-corer on the seafloor enhances gas release.
0:25	Gas release is enhanced when the multi-corer is triggered and cores are collected and recovered
